# Protein Complexes Form a Basis for Complex Hybrid Incompatibility

**DOI:** 10.3389/fgene.2021.609766

**Published:** 2021-02-09

**Authors:** Krishna B. S. Swamy, Scott C. Schuyler, Jun-Yi Leu

**Affiliations:** ^1^Division of Biological and Life Sciences, School of Arts and Sciences, Ahmedabad University, Ahmedabad, India; ^2^Department of Biomedical Sciences, College of Medicine, Chang Gung University, Taoyuan, Taiwan; ^3^Division of Head and Neck Surgery, Department of Otolaryngology, Chang Gung Memorial Hospital, Taoyuan, Taiwan; ^4^Institute of Molecular Biology, Academia Sinica, Taipei, Taiwan

**Keywords:** evolution, speciation, hybrid incompatibility, proteomics, proteins, bioinformatics

## Abstract

Proteins are the workhorses of the cell and execute many of their functions by interacting with other proteins forming protein complexes. Multi-protein complexes are an admixture of subunits, change their interaction partners, and modulate their functions and cellular physiology in response to environmental changes. When two species mate, the hybrid offspring are usually inviable or sterile because of large-scale differences in the genetic makeup between the two parents causing incompatible genetic interactions. Such reciprocal-sign epistasis between inter-specific alleles is not limited to incompatible interactions between just one gene pair; and, usually involves multiple genes. Many of these multi-locus incompatibilities show visible defects, only in the presence of all the interactions, making it hard to characterize. Understanding the dynamics of protein-protein interactions (PPIs) leading to multi-protein complexes is better suited to characterize multi-locus incompatibilities, compared to studying them with traditional approaches of genetics and molecular biology. The advances in omics technologies, which includes genomics, transcriptomics, and proteomics can help achieve this end. This is especially relevant when studying non-model organisms. Here, we discuss the recent progress in the understanding of hybrid genetic incompatibility; omics technologies, and how together they have helped in characterizing protein complexes and in turn multi-locus incompatibilities. We also review advances in bioinformatic techniques suitable for this purpose and propose directions for leveraging the knowledge gained from model-organisms to identify genetic incompatibilities in non-model organisms.

## Introduction

Reproductive isolation impedes gene flow between species or populations and is considered fundamental to speciation (Coyne and Orr, 2004). Genomes of diverging populations accumulate differences over evolutionary time. When such populations meet to form hybrids, they may suffer from genetic incompatibilities, which are detrimental to the hybrid populations. Genetic variants between the populations that are neutral and adaptive within the populations, but deleterious between the populations are known as Dobzhansky-Muller (DM) genetic incompatibilities (Dobzhansky, 1936; Muller, 1939). This type of negative epistasis between genetic variants can cause hybrid inviability or hybrid sterility and is an important driver of post-zygotic reproductive isolation (Presgraves, 2010a; Rieseberg and Blackman, 2010; Maheshwari and Barbash, 2011). Thus, the genes involved in DM genetic incompatibilities are commonly referred to as “speciation genes.” Although considerable effort has been devoted to identify speciation genes across several taxonomic lineages including fungi, animals, and plants, detailed molecular mechanisms underlying the failures in interactions between loci have been characterized in only a handful of cases (Wittbrodt et al., 1989; Ting et al., 1998; Brideau et al., 2006; Bomblies et al., 2007; Chang and Noor, 2007; Lee et al., 2008; Mihola et al., 2009; Brideau and Barbash, 2011; Chen et al., 2016a; Davies et al., 2016). This underlines the confounding effects of processes such as drift and linked selection, which can produce signatures similar to divergence, and also the complex genetic basis of reproductive isolation (Maheshwari and Barbash, 2011; Wolf and Ellegren, 2017). Furthermore, it should be noted that DM genetic incompatibilities are not necessarily interactions between proteins; but can also arise through the interactions of proteins with non-coding regions.

From some of these studies, there is evidence for rapid evolution in these speciation genes. Co-evolution between a rapidly evolving gene (with important function) and its partner loci are sometimes due to molecular arms races containing bouts of positive selection that can lead to the formation of speciation genes (Chou et al., 2010; Johnson, 2010; Presgraves, 2010a; Maheshwari and Barbash, 2011). One major category of such speciation genes are cyto-nuclear incompatibilities (Burton et al., 2013). Several theoretical models and experimental evidence support this class of incompatibilities across several species (Chou and Leu, 2010; Telschow et al., 2019). In yeast, almost all the known cases of DM incompatibilities are mitochondrial-nuclear incompatibilities (Lee et al., 2008; Chou et al., 2010; Chou and Leu, 2010; Presgraves, 2010a; Hou et al., 2015, 2016; Jhuang et al., 2017). Cyto-nuclear incompatibilities are also commonly observed in plants and animals (Burton et al., 2013). In recent years, genomic conflicts have been suggested to play a vital role in the formation of genetic incompatibilities (Johnson, 2010). Several known incompatibilities in plants are driven by selfish elements leading to hybrid necrosis, which has been reviewed in detail (Rieseberg and Blackman, 2010; Chen et al., 2016a). In addition to strong intracellular incompatibilities, extracellular factors can enhance the deleterious effect of some hybrid incompatibilities.

Environmental selection, even in absence of geographic barriers, is a known impetus of reproductive isolation (Hou et al., 2015, 2016; Landguth et al., 2015). This is often observed during separation of incipient species, where weak DM incompatibilities begin to emerge. In fact, mitochondrial-nuclear interactions in *Saccharomyces cerevisiae* populations can explain a significant proportion of the phenotypic variances under diverse environmental conditions. The allelic interactions between mitochondrial and nuclear genomes may be co-adapted to specific ecological niches that the yeasts occupy. Disturbing such naturally occurring interactions leads to breakdown of within-environment mitochondrial-nuclear epistasis, which provides fitness advantages in certain environments (Paliwal et al., 2014). Like nuclear genetic variations, mitochondrial genetic variations are also a source of adaptive potential. In isogenic strains containing recombinant mtDNAs, multiple loci interact epistatically and are specific to some environmental conditions. Interruption of co-adapted mitochondria-mitochondria interactions causes fitness defects in these environmental conditions (Wolters et al., 2015, 2018). Some mitochondrial-nuclear incompatibilities in yeast become evident only under stress when the hybrids of obligate fermentative yeast are forced to respire in non-fermentative carbon sources (Hou et al., 2015, 2016). These weak incompatibilities observed between *S. cerevisiae* populations may eventually lead to strong incompatibilities similar to those existing between close relatives of this budding yeast (Chou et al., 2010; Chou and Leu, 2010; Hou et al., 2016; Jhuang et al., 2017). Hybrid necrosis in plants can also be condition specific. For example, while in some plant hybrids weakness becomes evident at high temperatures (Bomblies et al., 2007; Alcazar et al., 2009; Chen et al., 2013, 2014), other plants usually demonstrate hybrid breakdown at low temperatures (Traw and Bergelson, 2010; Hua, 2013).

In all of the above examples, there is a strong motivation to identify the specific molecular mechanisms that can give rise to hybrid incompatibilities leading to speciation events. The most-simplified DM model assumes that genetic incompatibilities are due to pairwise genetic interactions that contribute additively to hybrid breakdown between diverging lineages (Turelli and Orr, 2000; Welch, 2004). In fact, almost all of the well-studied DM incompatibilities happen to be two locus incompatibilities. Several theoretical models based on the holey adaptive landscape model and Fisher’s geometric theorem has shown that multi-locus DM incompatibilities can exist. These models predict the fitness effects of mutations on a population based on the probability that allelic interactions are incompatible, and by estimating the effect of mutations on multiple traits (Fraisse et al., 2016). Experimental crosses have frequently shown that reduction in fitness can be due to impaired interactions at more than two locus (complex epistasis) supporting the theoretical predictions (Moyle and Nakazato, 2009; Kao et al., 2010; Burkart-Waco et al., 2012; Corbett-Detig et al., 2013). However, very few examples of complex epistasis have been dissected at the genetic level (Moyle and Nakazato, 2009; Tang and Presgraves, 2009; Kao et al., 2010; Corbett-Detig et al., 2013; Phadnis et al., 2015).

Another theoretical model explaining the advent of complex epistasis is the “snowball” effect. As populations diverge the number of incompatibilities are expected to increase faster than linearly, “snowballing” the populations toward distinct species (Orr and Turelli, 2001; Turelli and Moyle, 2007; Presgraves, 2010b). Testing the “snowball” effect requires information on genetic incompatibilities between species at different divergence times. Since this information is hard to get, evidence for the “snowball” theory has been scarce. However, genetic mapping data in *Drosphila* (Matute et al., 2010) and *Solanium* species (Moyle and Nakazato, 2009) has demonstrated that accumulation of weak DM incomaptibilites can “snowball” and strengthen the genetic barrier between species. The original “snowball” model (Orr, 1995) was developed for diverging populations, considering simple DM incomaptibilites, and did not account for loss of DM incomaptibilites when these populations diverged. Later extensions of this “snowball” model (Kalirad and Azevedo, 2017) has shown that DM incomaptibilites do not necessarily function independently of each other and several DM incomaptibilites can arise and then disappear, while the populations are diverging, more so in complex incompatibilites (more than two loci) that are derived from the changes subsequent to the intial DM incompatibilities.

Factors that modulate the effect of two or more incompatible locus are allele frequency and genetic background. While changes in allele frequencies at incompatible locus can affect its relationship with its partner locus, a change in genetic background can alter the strength and magnitude of the incompatible locus (Wade, 2002; Johnson, 2010). Thus, individually some components of complex epistasis are either weak or they demonstrate incomplete penetrance (Lopez-Fernandez and Bolnick, 2007), but are synergistic, and demonstrate severe fitness defects and cause hybrid breakdown, when all the members of complex epistasis are present (Wu and Palopoli, 1994; Presgraves, 2010a; Lindtke and Buerkle, 2015; Schumer et al., 2015). As discussed earlier, these weak incompatibilities formed at the early stages of speciation may initially reduce gene flow between populations and “snowball” to become strong incompatibilities. Thus, deciphering complex epistasis can yield insights into the process of divergence at the early stages of speciation and are important for determining the rate and patterns of evolution during speciation (Kondrashov, 2003; Welch, 2004; Maheshwari and Barbash, 2011).

## Protein Complex Microenvironments Provide a Molecular Basis for Multi-Locus Incompatibilities

**Figure 1:**
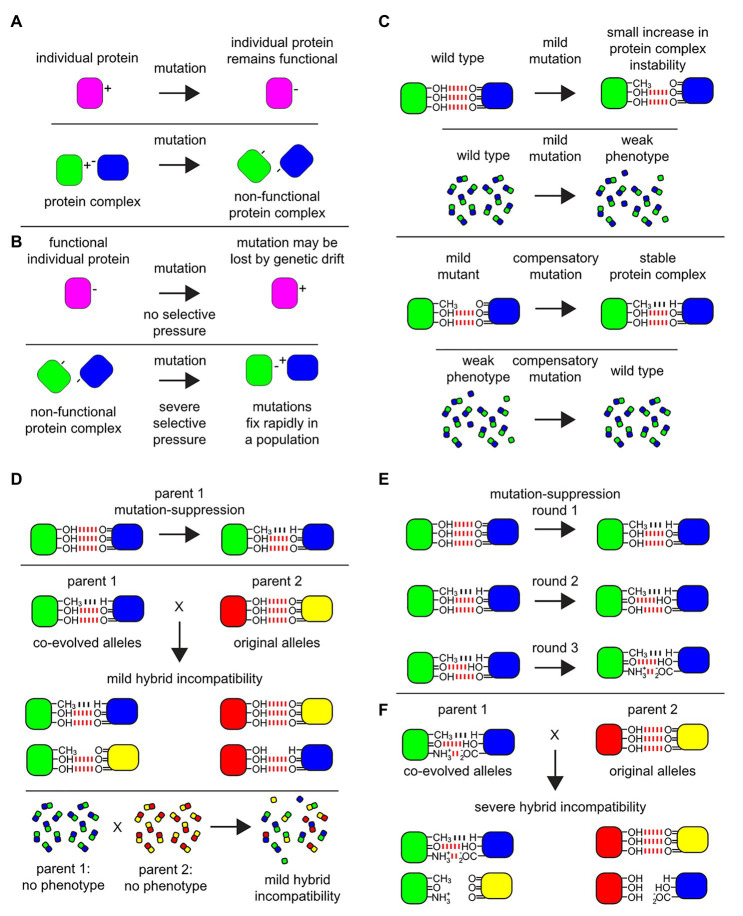
Protein complex micro-environments provide a molecular basis for multi-locus incompatibilities. Among biomolecular physical interactions within cells that give rise to hybrid incompatibilities, protein-protein interactions (PPIs) are a rich target for experimental investigation. **(A)** An example of a mutation on a monomeric protein verses a similar mutation at the interface of a PPI. A monomeric protein (magenta) undergoes a mutation at a surface residue from a positively charged amino acid residue to a negatively charged one which displays no phenotype and is fully functional. By contrast, in a protein-protein complex (green-blue) a mutation in one of the subunits (green) at a surface amino acid residue within the interaction domain from a positively charged residue to a negatively charged one could cause a loss of function, which may display a phenotype subject to selective pressure. **(B)** The mutation in the monomeric protein may be lost over time due to genetic drift. By contrast, among the subunits of the non-functional protein-protein complex, under severe selective pressure, a compensatory mutation may occur in the binding partner protein (blue) to allow for the formation of a function complex where both mutations rapidly fix within the population. However, note that the charge residues on the surface binding sites have been reversed between the subunits. **(C)** An example of a mild mutation within a protein-protein interaction site. An interaction between subunits in a multi-protein complex are stabilized by non-covalent bonds, where in this example three hydrogen bonds (red) create the binding energy to stabilize the complex. The fictional mutation illustrated here leads to the loss of one hydrogen bond in the protein-protein interaction domain of the complex. Within the cell, a mild mutation may lead to a small decrease in the average stability of the protein complex, illustrated in this diagram as a failure of only a few protein complexes to maintain their structure, yielding a very mild phenotype. Under selective pressure over time, compensatory mutations can occur in the binding partner to suppress the mild defect and fix within the population (bottom), illustrated here as a Van der Waals potential (black). **(D)** Co-evolved binding partner alleles can be uncovered as incipient mild hybrid incompatibility, which may or may not give rise to a detectable phenotype in hybrids. Even if there is no detectible phenotype, at the quantitative level among the population of individual proteins and protein complexes within the hybrid cells, this incipient incompatibility can lead to failures in the proper assembly of the protein complex and/or lead to a decrease in the stability of the protein complex (bottom). **(E)** After multiple rounds of mutations that display a mild phenotype, followed by compensatory suppressor mutations in the binding partner, multiple changes in the micro-environment in the protein-protein interaction domains can accumulate and fix in the population. **(F)** The fixation of these mutations in the protein-protein interaction domains can be revealed later as a source of hybrid incompatibility.

Proteins, the building blocks of the cell, execute many cellular functions through protein-protein interactions (PPIs). A large fraction of PPIs in eukaryotic proteomes culminate as heteromeric-protein complexes, and are responsible for diverse biochemical activities essential to cellular homeostasis, growth, and proliferation. For example, over 60% (about 3,600 proteins) of the *S. cerevisiae* proteome (Pu et al., 2009; Benschop et al., 2010; Costanzo et al., 2016), over 7,700 proteins in humans (Drew et al., 2017a), and over 2,700 proteins in fly (Guruharsha et al., 2011), are identified as subunits of protein complexes. Amino acid residues can evolve at different rates within a protein, with compensating mutations co-evolving at protein interaction sites (Zhang and Gu, 1998; Neverov et al., 2020; Figures 1A,B). The residues at intra-protein contact sites also co-evolve with entangled substitutions. A mildly destabilizing mutation at a protein site may lead to a compensatory mutation at its contact site that is fixed more easily and re-establishes the protein’s function (Figures 1C,D; Weigt et al., 2009; Burger and van Nimwegen, 2010; Morcos et al., 2011; Rizzato et al., 2019). This leads to variability in accumulation of substitution types (heterotachy and heteropecilly), becoming more evident as the species diverge (Figures 1E,F; Lopez et al., 2002; Roure and Philippe, 2011). Such micro-evolution within a protein can lead to co-evolution of partner proteins both in pairwise protein interactions as well as protein complexes. With individual subunits within a protein complex often sharing similar evolutionary patterns, alterations in partner proteins can decrease stability of protein complexes, create the loss of interactions, and even lead to failures in their assembly (Harrison and Burton, 2006; Juan et al., 2008). Protein-protein interactions are one of the major determinants of protein evolutionary rates and in general evolutionary rates are negatively correlated with the number of interactions (Fraser et al., 2003; Fraser and Hirsh, 2004; Fraser, 2005). Also, physically interacting proteins tend to co-evolve, and are precisely co-expressed to maintain the proper stoichiometry of interacting partners (Fraser et al., 2004). However, there are several known protein complexes with complex subunits (within conserved complexes) that still show high evolutionary rates deviating from the general trend. An example of protein complexes with such contrasting patterns of evolution would be the nuclear pore complex and RNA polymerase II (Leducq et al., 2012). Even though the underlying driving force is unclear, these proteins are the candidates that may cause incompatibilities.

Co-evolving protein-protein interactions depend on the phenotypic traits, which are species specific and maintained by reciprocal selection (Brodie and Ridenhour, 2003; Klink and Bazykin, 2017). These principals of protein co-evolution have been exploited to predict protein-protein interactomes at a genome scale and also in protein structure predictions (Marks et al., 2012; Hopf et al., 2014; Anishchenko et al., 2017; Cong et al., 2019). Thus, the divergence of orthologs proteins between species or populations can manifest as incompatibilities in hybrids, when co-evolving partner proteins gets shuffled/swapped from the two parents in the hybrids (Clark et al., 2009; Tang and Presgraves, 2009; Zamir et al., 2012; Ochoa and Pazos, 2014). Furthermore, proteins often fold into functional conformation only after interacting with their partners (Dunker et al., 2008). Loss or failure in interactions can lead to protein misfolding and loss of its stability. Interacting partner proteins thus tend to co-evolve with each other within a species (Neverov et al., 2020) and are also often co-expressed with one another, to maintain proper stoichiometry among interacting components (Ge et al., 2001; Fraser et al., 2004). This is more evident in protein complexes, as multiple proteins are required to interact, and the functional folded conformation of protein complexes may depend on some of these interactions.

## Mis-Assembly of Protein Complexes can be a Source of Multi-Locus Incompatibilities

**Figure 2:**
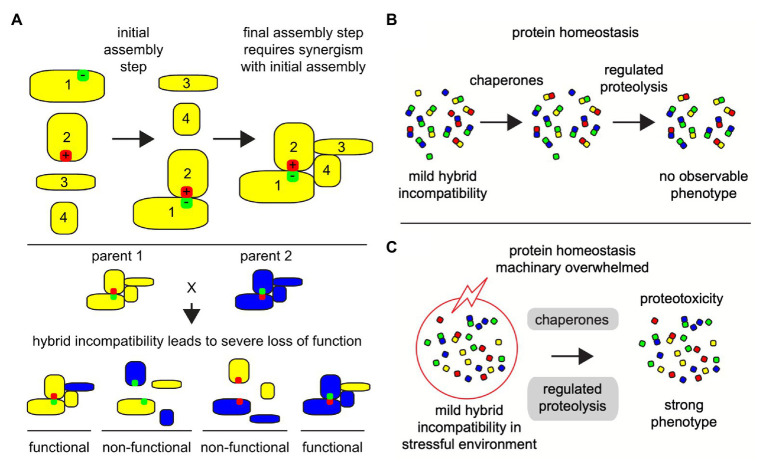
Co-evolving protein-protein interactions depend on the phenotypic traits and stressful environmental and cellular conditions can reveal hybrid incompatibility phenotypes. Illustrated examples of protein micro-environment in a protein-protein complex structural and functional integrity. **(A)** Large multi-subunit protein complexes are assembled in a step-by-step manner, where hybrid incompatibility may lead to the loss of the entire functional complex, especially if one of the evolved binding partners is necessary for an early assembly step. In this illustration protein 1 and protein 2 must assemble first in order to promote a stable interaction with proteins 3 and 4. If mutation-suppression occurs in divergent organisms, such as the reverse of positively (red) and negatively (green) charged amino acid residues as illustrated here, then a severe disruption in the protein complex assembly can occur (bottom). **(B)** Under congenial environments, when cells are challenged by mild forms of intrinsic protein-protein incompatibilities, the cellular homeostasis machinery, including chaperones and regulated proteolysis, protects the cells and promotes the formation and selection of functional protein complexes. **(C)** Even under weak stressful environments (red circle with lightning), mild protein-protein incompatibilities can accentuate to higher levels of unfolded and mis-assembled proteins, leading many cellular complexes to fail and overwhelm the protein homeostasis machinery (gray) causing a collapse in cellular protein homeostasis and proteotoxic stress.

Most protein complexes are assembled co-translationally, unidirectionally (Gloge et al., 2014; Shiber et al., 2018; Schwarz and Beck, 2019) and in a specific order (Marsh et al., 2013). Protein complex assembly is a multistep process and must proceed *via* the most energetically optimum path of bringing together proteins to form intermediate subcomplexes, which is further extended to form complete protein complexes (Figure 2A). By analogy to Levinthal’s paradox of protein folding (Levinthal, 1969), just as proteins fold *via* limited number of energetically favorable folding pathways, protein complexes should be expected to assemble following a strict order and known to be under evolutionary selection (Marsh et al., 2013). Deviation from the energetically optimum order can lead to mis-assembly of proteins with severe biological consequences (Dobson, 2003; Ellis, 2007). The assembly and maturation of protein complexes are coordinated by molecular chaperones. Generally, protein homeostasis machinery and related chaperones closely monitors and prevents the formation of misfolded protein aggregates (Asher et al., 2006; Hartl et al., 2011), protein trafficking, and enzyme activity regulation (Figure 2B; Mashaghi et al., 2016). The chaperone proteins require the help of co-chaperones and assisting proteins in assembling the protein complexes (Marsh and Teichmann, 2015). One of the ubiquitous family of chaperones important for protein folding and protein complex assembly are the heat shock proteins (HSPs). Chaperoning and assembly of protein complexes by HSPs has been studied in considerable detail (Mcclellan et al., 2007; Li et al., 2012; Makhnevych and Houry, 2012; Gopinath et al., 2014; Marsh and Teichmann, 2015). First, Hsp70, Hsp40, and client proteins form an early complex, and this is transferred to Hsp90 with the help of the adaptor protein Hop/Sti1 for correcting late stage misfolding, and final assembly.

In hybrids, which are the outcome of the mating of parents from related species, the protein complexes are formed from an admixture of subunits from the two parents. Under congenial environmental conditions, the homeostasis machinery in hybrids derived from closely related species or populations works toward maintaining the protein-protein interactions and protein complexes, at least in a partial functional state (Figure 2B; Leducq et al., 2012; Piatkowska et al., 2013; Zhong et al., 2016). However, when these closely related hybrids are exposed to even mildly harsh external environments, the protein homeostasis machinery is overburdened and leads to breakdown of interaction between proteins (Figure 2C). This has been evidenced in several studies (Leducq et al., 2012; Zill et al., 2012; Piatkowska et al., 2013). Mis-assembly of protein complexes due to failures in protein-protein interactions can impair the protein homeostasis machinery invoking a proteotoxic stress response, and can cause severe growth defects (Gidalevitz et al., 2011; Arslan et al., 2012; Oromendia and Amon, 2014; Radwan et al., 2017). This is also true for essential proteins. Several essential proteins are subunits of protein complexes and are incompatible as chimeric assemblies from closely related species (Lai et al., 2018). Proteotoxic response in hybrids could be due either or all of the above-described reasons, i.e., loss of interactions among co-evolving proteins, reduced protein expression, mis-folding of proteins to non-functional configurations. Protein complexes involve multiple interactions and can result in different levels of functional defects depending on how many interactions are compromised. Thus, phenotyping chimeric protein complexes in parental hybrid cellular environments can enhance our insight into the molecular basis of emergence and fixation of multi-locus incompatibilities. This requires determining and analysis of large-scale proteomics data in parental species as well as their hybrids.

In addition, mis-regulation of protein complexes in hybrids could also be due to transcriptional mis-regulation or mRNA instability (Supplementary Figure 1). Divergence between interacting regulatory elements or regulatory divergence is another common route through which DM incompatibilities can arise (Mack and Nachman, 2017). mRNA abundance is regulated by the binding of *trans*-factors (mainly Transcription Factors) to *cis*-regulatory elements, which are short stretches of non-coding DNA, thus mutations on either of them can affect the mRNA abundance of target genes. While *trans*-factors are known to be under higher selection constraint than their *cis*-counterparts, they can evolve faster than other classes protein coding genes (Castillo-Davis et al., 2004). Transcriptional regulation diverges quickly between closely related species that often leads to mis-regulated gene expression in hybrids (Landry et al., 2005; Tirosh et al., 2009; Tirosh and Barkai, 2011; Swain Lenz et al., 2014; Mack and Nachman, 2017; McGirr and Martin, 2019). Although, inter-species transcriptomic analysis has shown that changes in transcript levels are frequently deleterious (Gilad et al., 2006), gene regulatory networks are not necessarily conserved between species (True and Haag, 2001). The evolution of gene expression can be explained under a “house of cards” model of stabilizing selection, where mutations affecting mRNA abundance can lead to a deluge of changes between co-evolved *cis*-elements and *trans*-elements in an evolutionary network. Such evolutionary cascade has been observed in yeast, worms and flies (Hodgins-Davis et al., 2015). Further details on the theoretical and empirical considerations of gene regulation and their implications on speciation can be found in a recent review by Mack and Nachman (2017).

Promoter-mediated coupling of transcription to mRNA degradation is known to be diverged between closely related species (Dori-Bachash et al., 2012). Altered rates of mRNA degradation can impact dosage of available transcripts for translation affecting protein expression and abundance. The translational efficiency of proteins also changes with alterations in intrinsic and extrinsic environmental conditions. One of the mechanisms by which the translational machinery adapts to such changes is by modulating their tRNA usage (Meiklejohn et al., 2013; Yona et al., 2013; Bloom-Ackermann et al., 2014). The translational efficiency is dependent on cellular concentration of tRNA molecules and the efficiencies of each codon-anticodon pairing (Dos Reis et al., 2004). There is an apparent connection between tRNA availability and protein folding. Since, tRNA usage is fine-tuned within each species, changes in tRNA pool in interspecies hybrids can affect their translational efficiency and protein folding (Meiklejohn et al., 2013; Yona et al., 2013; Bloom-Ackermann et al., 2014). When one or more subunits of a protein complex is mis-regulated due to anyone of the above reasons or their mRNA is degraded, dosage of available transcripts for it is altered for these subunits. This can interfere with the tight stoichiometry with which protein complex subunits are produced, leading to protein mis-assembly. Furthermore, DM incompatibilities due to regulatory divergence can also lead to breakdown in protein complexes. Protein complexes can be compared to a jigsaw puzzle, with each subunit forming an element of the puzzle. Misfolded or missing complex subunits might not be able to complete this puzzle leading to incomplete protein complex assembly.

## Proteome-Scale Analyses of Protein Complexes

Protein complexes are usually identified by characterization of PPI. Some of the commonly used experimental strategies to detect PPIs, include the yeast two-hybrid system (Y2H; Paiano et al., 2019), protein-fragment complementation assay (PCA; Tarassov et al., 2008; Michnick et al., 2010), fluorescence resonance energy transfer (FRET; El Khamlichi et al., 2019), and affinity purification plus mass spectrometry (AP-MS; Gavin et al., 2006; Krogan et al., 2006; Volkel et al., 2010; Babu et al., 2012; Adelmant et al., 2019). These methods help us collect a vast set of protein-protein interaction data from different organisms. However, most of these approaches are limited by the availability of high-quality antibodies or sequence-verified cDNA clones suitable for targeted protein complex enrichment, labor-intensive and not suitable for the global complex dynamic studies in which many time points and conditions are involved.

**Figure 3:**
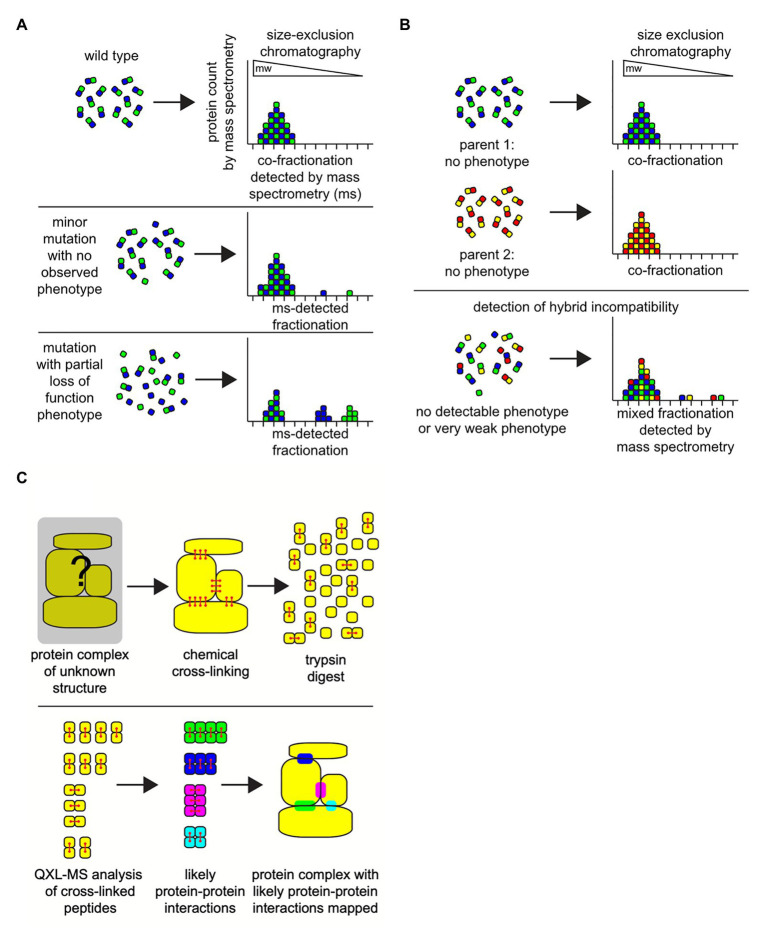
Combining column chromatography with mass spectrometry (MS) can successfully identify protein complexes. The integrity of protein-protein interactions in hybrids can be measured directly using classic protein column chromatography combined with ultra-sensitive MS techniques. **(A)** Under defined environmental conditions the spectrum of protein complexes in a cell can be characterized by employing a combination of cell extraction, size-exclusion chromatography (gel filtration), and MS analyses on the resultant fractions. Size-exclusion chromatography separates proteins and intact multi-subunit protein complexes based on their size (molecular weight; mw) and shape, where large-sized proteins or complexes elute from the column in early fractions (left), and smaller proteins elute in later fractions (right). Due to the sensitivity of mass spectrometry, even very small perturbations in the physiological states of protein complexes can be detected, even when there is no observable phenotype associated with the protein complex function in the organism (middle). Or, observable mutant phenotypes based on protein-protein interactions that fail can be detected as large bio-signatures of unassembled protein complex subunits (bottom). In this example, only one protein-protein complex is illustrated. In a biological cellular extract sample, there will be 1,000 s of overlapping protein complexes of various sizes and shapes in each fraction, all of which can be detected in unison *via* mass spectrometry. **(B)** In hybrids, size exclusion chromatography-mass spectrometry analyses can reveal evidence for weak incipient hybrid incompatibilities that do not display a severe phenotype (bottom). **(C)** Identifying and mapping the location of protein-protein interactions domains on the surfaces of proteins may identify residues that can contribute to hybrid incompatibilities. Crosslinking reagents chemically react with physically close and exposed amino acid residues on the surfaces of protein binding pairs in a complex (red). These covalent crosslinks are maintained during proteolytic digestion of the proteins with the enzyme trypsin in preparation for mass spectrometry. The cross-linked peptide-peptide fragments will be detected as a single molecule during mass spectrometry (bottom). Analysis of the collection of cross-linked fragments in a sample can be employed to create a physical map of a likely protein-protein interaction domain, a region that will have the potential to contribute to hybrid incompatibility within the potential protein-protein interaction sites in a protein complex.

Recent advances in mass spectrometry techniques allow us to develop an effective approach to quantitatively measure PPIs. To gain a global view of the complex behavior, transient and higher-order associations of the proteome under a state mimicking the native cellular condition needs to be captured. Some of the recent studies have successfully shown that cellular fractions could serve as a proxy for the cellular environment and retain basic cellular organization especially for the non-membrane proteins (Kristensen et al., 2012; Kastritis et al., 2017; Figure 3A). Such approaches have also proven to be effective in non-model organisms. A recent study combined size exclusion chromatography and ion-exchange chromatography with tandem mass spectrometry to successfully identify protein complexes in five metazoan species *Caenorhabditis elegans* (worm), *Drosophila melanogaster* (fly), *Mus musculus* (mouse), *Strongylocentrotus purpuratus* (sea urchin), and human. The identified protein complexes were compared with the data derived using a similar co-fractionation strategy in *Xenopus laevis* (frog), *Nematostella vectensis* (sea anemone), *Dictyostelium discoideum* (amoeba), and *S. cerevisiae* (yeast) to gain insight into biochemical evolution of protein interactions and conservation of protein complexes in species diverged by over billion years (Wan et al., 2015).

Although the co-fractionation strategy by itself has been found to be effective, it still lacks sensitivity for in depth analysis of protein complex dynamics. The sensitivity in identification of proteins and its complexes can be improved significantly with stable isotope labeling by amino acids in cell culture (SILAC; Ong et al., 2002). By incorporating the SILAC protocol, samples from different conditions or time points can also be accurately quantified and compared. Since SEC separates the protein complexes based on their size, the subunits of an assembled complex should be observed in the same fractions (Kastritis et al., 2017). If the same complex subunits appear in different fractions under different condition, it indicates that the complex structure has been altered or destabilized (Figure 3A). There are only a handful of studies that have attempted to determine protein complexes in hybrids (Figure 3B; Leducq et al., 2012; Piatkowska et al., 2013; Bontinck et al., 2018). Most of these studies have focused on specific protein complexes in related species. An experimental limit of the SILAC approach is that cells or organisms need to be cultured in the isotope-containing medium before the samples are prepared, thus non-applicable to the systems that cannot be cultured in the lab. In recent years, the efficiency of isobaric labeling methods, such as tandem mass tags (TMT), have been greatly improved (Thompson et al., 2003; Wang et al., 2020).

Although the *in vitro* labeling process inevitably introduces more variation into the samples, it provides an alternative approach for accurate measurements of protein complex dynamics in unculturable systems. The co-fractionation approach to detect the potential basis of complex hybrid incompatibility is also limited by the need for exceptional rigor and caution during sample preparations, as the signature for incompatibility is the disruption of protein-protein interactions as detected by SEC/MS, which can also easily result from mis-handling of samples. In general, very small differences in sample preparations and sample handling can lead to disparate results, where it remains unclear which sample(s) contain the most accurate representation of the spectrum of protein complexes *in vivo* (Ho et al., 2002). One set of fundamental unavoidable physical variables contributing to these uncertainties is simply that the act of cell lysis always dilutes the concentration of the proteins into a new solution environment that does not precisely match the native cytosol, where this disruption in protein complex homeostasis is also amplified by the inherent errors in human liquid handling through time, and on milliliter or microliter scales. Automated nano-volume high-precision rapid cell extraction may help to suppress variation in sample preparation. Toward this end, one promising technical development is nano-scale single-cell mass spectrometry technologies, where, in the future, it may be possible to couple these nano-liquid-handling approaches with a size-fractionation step in order to map out the distributions of protein-protein interactions with high-accuracy and reproducibility (Williams et al., 2020). Alternatively, before cell extraction, or immediately upon cell extraction, covalent chemical cross-linking can be employed to both stabilize protein-protein interactions and to potentially map interaction domains, but has the drawback that non-specific cross-linking can occur leading to false-positive results.

In general, co-fractionation mass spectrometry (CF-MS) can only be used to infer protein complexes when the information of protein functions or interactions is available. On the other hand, quantitative cross-linking mass spectrometry (QCLMS/QXL-MS) can be used to directly detect protein interaction sites and binding partners (Figure 3C). Reactive groups in proteins and protein complexes are cross-linked and subjected to mass spectrometry. The mass spectrometric signals of cross-linked peptides derived from different conformations can then be distinguished. The location of the cross-links inflicts a distance constraint on the respective side chains orientation and position. Thus, it can be used to draw conclusions on the three-dimensional structure of the protein or topology of a protein complex (Sinz, 2006; Chen et al., 2016b; Kastritis et al., 2017; Chen and Rappsilber, 2019). The recent advances of QXL-MS allows us to construct the global protein complex list in the organisms for which information of protein functions or interactions are limited (Chavez et al., 2019). Moreover, it can be used to measure the change of protein-protein interaction systematically or detect chimeric protein complexes in hybrids.

Current high-throughput proteomics platforms come with several software tools for pre-screening, identification, assembly, and quantification of detected peptides. Such built-in tools are usually tailored for optimization of the data generated by mass spectrometers depending on their fragmentation processes and collision energies and generally do not work across platforms. MASCOT, OMSSA, SEQUEST, X!Tandem, and TOPPAS are some of the popularly used platforms for analyzing tandem mass spectrometry data. The popular tools that are used for analysis of peptides generated from SILAC or QCLMS/QXL-MS followed by LC-MS/MS are Census, MaxQuant, MsQuant, MASCOT Distille, and COFRADIC (Kohlbacher et al., 2007; Nesvizhskii et al., 2007; Cox et al., 2009; Deutsch et al., 2010; Nahnsen et al., 2011; Doerr, 2012; Kristensen et al., 2012; Yates et al., 2012). One of the major draw backs of proteomics data is that the signal to noise ratio is extremely small. The tools developed till-date have been developed to enhance the signal and reduce the noise in the data. Filtering noise and identifying species specific proteins in proteomics data from hybrid species, especially from closely related species, is challenge, leading higher false positive rates in the predictions. Fractionation based on size and isotope labeling of amino acids followed by tandem mass spectrometry has improved the resolution of separation of peptides, but there is still significant loss of data in hybrids due to lack of specificity in peptides between the two species forming the hybrid. However, in the recent years, considerable effort has been devoted to improving the experimental and analysis techniques of mass spectrometry and the limitation of proteomics can be expected to be alleviated in the near future. This is corroborated by several studies in the last few years, where global analysis of protein complexes has been done in a wide range of higher order biological systems including plants (Aryal et al., 2014), pluripotent stem cells, and cancer cells (Sudhir and Chen, 2016). The technology has also advanced to handle large scale quantitative proteomics; proteins in 375 cancer cell lines were quantitatively profiled recently using mass spectrometry (Nusinow et al., 2020). Developing experimenting techniques and the analysis software that is specifically applicable to hybrid analyses is an area of research with definitive scope for innovation and development.

## *In Silico* Prediction of Protein Complexes

Almost all the available methods for predicting protein complexes rely on protein-protein interactions. Experimental data are the primary source of these protein-protein interactions. Existing prediction methods can be classified into network-based approaches and functional information-based approaches. Although, methods from the two categories are used independently to predict protein complexes, they are also frequently used in combination (Price et al., 2013; Srihari and Leong, 2013; Zahiri et al., 2020). The network-based approach classifies protein-protein interactions as protein complexes based on the density of protein interactions, and topology of network structure. The functional information methods, also known as biological context-based methods, supplements the protein interaction network with information from functional annotation such as gene expression, gene ontology, and protein domain architecture. Supplementing annotation is known to improve the accuracy of predictions more than those based only on pure protein network approaches (Price et al., 2013; Srihari and Leong, 2013; Zahiri et al., 2020).

Accurate predictions of network topologies require accurate affinity scoring schemes. The most accurate whole organism complexome is derived from high-throughput TAP-MS studies, based on the affinity scoring schemes developed specific to their experimental design and output (Gavin et al., 2006; Krogan et al., 2006; Collins et al., 2007; Hart et al., 2007). The affinity scores can be used to determine the confidence and reliability of observed protein-protein interactions. Availability of accurate affinity scoring schemes from TAP-MS data is currently limited to a few standard model organisms such as yeast *S. cerevisiae*. In higher order organisms and non-model species direct protein-protein contacts can be derived using the from the covariation pattern of the protein abundances from CF-MS data. The three-dimensional arrangement of protein contacts leading to protein complexes can be determined by performing clustering analysis of correlation matrices derived from CF-MS profiles using machine learning algorithms. The CF-MS system has been successful in identifying complexomes of *Caenorhabditis elegans*, human cell lines, and 13 plant species of agricultural and scientific importance (Drew et al., 2017b; Hu et al., 2019; McWhite et al., 2020). The CF-MS correlation profiles can be considered analogs to affinity scores from TAP-MS (henceforth referred to as affinity scores for simplicity). Markov Clustering algorithms (MCL) have proven to be reasonably successful as a method for incorporating information from multiple resources and developing weighted protein interaction networks (Srihari and Leong, 2012). MCL is an unsupervised cluster algorithm, which simulates a series of random walks and iteratively computes the probability that the cluster of protein interactions is dense or sparse during each visit (Enright et al., 2002; Van Dongen and Abreu-Goodger, 2012). In unsupervised clustering algorithms, the data is not labeled and the model is allowed to work on its own to group data inherently into clusters. When the probability of cluster is dense, the MCL will not leave the cluster. Based on the thickness and spread of the random walks to a cluster, MCL identifies protein complexes.

The basic MCL works well for small clusters but is not successful for clusters with more than 10 partners. This bias to prediction of several small clusters (fragmentation bias) has been resolved to a large extent by advance variants of MCL such as Multi-level regularized MCL (Satuluri and Parthasarathy, 2009; Satuluri et al., 2010) and Parallel Shotgun Coarsened MCL (Lim et al., 2019). These methods start with initial clusters using MCL and then build bio-networks of refined clusters by incorporating core-attachment structures to generate complexes. Detecting core-attachment happens in two stages (Wang et al., 2019). First, a subgraph with maximum clique, i.e., largest complete subgraph with all its vertices connected to each other, are identified as a protein core. Next, protein attachment structures are determined by selecting proteins that interact with more than half of the neighboring protein cores.

Despite these advances, the false positive rate in protein complex prediction is high. This is mainly due to high variability in complex core sizes and the sparsity of protein-protein interaction networks. Also, the protein complex compositions can vary between experiments for the same strains of the same species. These differences can be due to the experimental procedures, dissimilar coverage in data from different experiments and from the application of pre-screening software. Thus, it can lead to an ambiguity on the correct composition of protein complexes. To overcome these limitations, studies have tried to include functional information of interacting proteins. This information includes protein domain architecture, protein expression, gene expression, and gene ontology. Recent methods have indicated that including evolutionary information and genetic algorithms can improve the accuracy in protein complex predictions (Zahiri et al., 2020). Bayesian networks and other probabilistic classifiers developed using the parameters derived from MCL and its variants along with the functional parameters have also been found to be reliable (Gavin et al., 2006; Krogan et al., 2006; Collins et al., 2007; Hart et al., 2007; Wang et al., 2009a). These classifiers are also useful in determining protein complexes from protein contacts derived from CF-MS data. Although, evolutionary algorithms and Bayesian classifiers are more accurate, they are computationally expensive. While, considerable work has been devoted to optimizing Bayesian classifiers, evolutionary algorithms although superior in their predictions, are yet to be optimized for efficient application on large networks.

Furthermore, considerable effort has been devoted to develop algorithms for compiling core consensus protein complexes from diverse data resources. For example, Benschop et al. (2010) developed a forward-backward module detection algorithm that predicts consensus of protein complexes from AP-MS data in *S. cerevisiae*. This algorithm searches across the diagonal on the clustered protein interactome matrix for strongly interacting proteins, by traversing first from right to left and then from top to bottom. This is the forward phase of the algorithm, in the backward phase the direction of traversal is reversed, and detects and accounts for non-interacting proteins. Similar methods have also been developed for predicting consensus soluble protein complexes isolated from human HeLA S2 and HEK93 cells (Havugimana et al., 2012) and *Arabidopsis thaliana* (Gorka et al., 2019).

Bioinformatics methods have also played an important role in advancing the field of proteomics into more complex areas. For example, the determination of composition of membrane protein complexes, have been elusive as upon employing detergents, which is necessary to elute membrane proteins, protein complexes fall apart. Recent studies have determined the protein complexes based on the protein interaction maps in *Escherichia coli*, *S. cerevisiae*, and human mitochondria (Babu et al., 2012; Malty et al., 2017; Babu et al., 2018). They have also helped in determining the biochemical evolution of protein complexes diverged by over a billion years (Wan et al., 2015). Together, these demonstrate the importance in advancement of bioinformatics tools and algorithms in the field of proteomics and predicting protein complexes. However, most of the currently available bioinformatics tools are designed for pure species and mainly tuned to work efficiently on data from a handful of model organisms. The underlying reason is that the availability of proteomics and protein-protein interaction data is also limited to this subset. Thus, there is a huge void in both data and techniques to deal with non-model organisms, and, more importantly, hybrid organisms.

## Genomes and Transcriptomes can Assist Predicting Genetic Incompatibilities in Non-Model Species

Next Generation Sequencing (NGS) technologies have the potential to work with any species, determine the natural variation at a genome-wide level and at unprecedented resolution, and also can provide us to with a comprehensive picture of regulatory variation. Over the past decade, such genomic technologies have been used to explore the extent of natural variability at the molecular level and the evolutionary forces that shape this variation (Gilad et al., 2009; Wolf et al., 2010). In several cases, patterns of variation at either structural or regulatory levels have helped in explaining physiological and morphological phenotypes (Hoekstra and Coyne, 2007; Carroll, 2008). NGS has also been able to discover genetic incompatibilities in *taxa* at their incipient stage of speciation and introgressed species (Barreto et al., 2011) as well as non-model species (Gagnaire et al., 2012).

Resequencing and *de novo* assembly are two frequently used modes of genome assembly. Resequencing refers to sequenced reads performed when a reference genomes are available and *de novo* assembly is used when the reference genome is of poor quality or when no reference genome is available. *De novo* assembly is more computationally intensive compared to resequencing but has wider applications. The quality of a genome assembly provides a measure of the degree to which the sequence has been correctly assembled and the sequences are reliable, and thus of great importance. Assembly quality can be assessed using different statistics, which offer a measure of genome completeness and contiguity (Yandell and Ence, 2012; Lachance and Tishkoff, 2013; Simao et al., 2015; Liao, 2019). Excellent reviews are available on *de novo* genome assembly (Liao, 2019) and use of genome sequencing in non-model organisms (Ellegren, 2014). The recently developed third generation sequencing technologies can produce extra-long reads with a median length of 10–20 kb and sometimes longer than 50 kbp. Such long reads can facilitate better quality of *de novo* assemblies especially in non-model organisms (Giani et al., 2020). Similarly, in RNA-Seq reads can be assembled *de novo*, as well as mapped to a reference genome or transcriptome and used to quantify gene expression changes between the organisms of interest (Wang et al., 2009b; Oshlack et al., 2010). In addition, transcriptomic data from RNA-Seq can also be used for population genetic analyses, test for selection or isolate SNPs/microsatellite markers for further population genetic or genetic mapping studies (De Wit et al., 2012; Sun et al., 2012).

**Figure 4:**
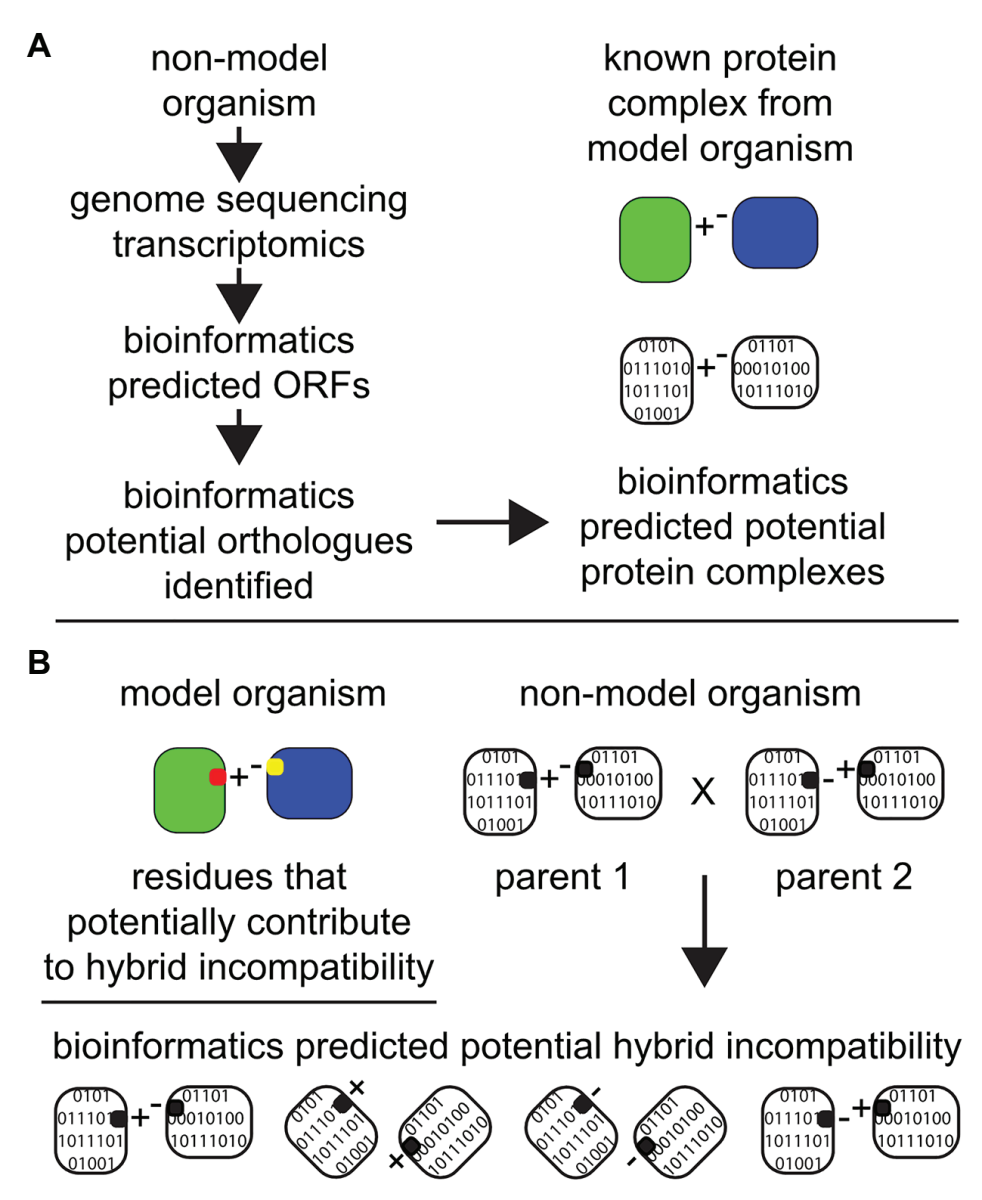
The importance of advances of bioinformatics tools and algorithms in the field of proteomics and in predicting protein complexes. Non-model organisms can be explored *in silico* after the completion of genome sequencing and/or transcriptomic analyses with the application of bioinformatics. **(A)** Databases from model organisms can be employed to make predictions about potential protein complexes in non-model organisms. Based on predicted ORFs from genomic sequencing and/or transcriptomics, orthologs proteins can be identified in non-model organisms and “assembled” *in silico* to predict the potential existence of a protein complex in the unstudied system. **(B)** Amino acid residues that have been established or predicted to contribute to hybrid incompatibility in the model organism (represented as red and yellow) are then employed as a target for analyses to investigate the potential for hybrid incompatibility within two related non-model organisms with the potential to make hybrids.

Current genetic and molecular biology strategies are confined to two or three locus incompatibilities due to practical and technical restraints. Determining multi-locus incompatibilities is difficult due to the high level of epistasis in hybrids (Kao et al., 2010; Li et al., 2013). Since phenotypic traits due to genetic interactions (epistasis) can appear in ratios deviating from those expected with independent assortment, it becomes more difficult to detect multi-locus incompatibilities. Predicting and validating multi-locus incompatibilities requires considerably larger sample sizes and is a major hurdle in traditional approaches. Transcriptomic and genomic information can help to generate possible protein interaction networks that can further be used to alleviate the complexity of epistasis (Figure 4; Angeles-Albores et al., 2018). There are a few reviews elaborating the importance of integrative bioinformatic approaches to incorporate genomic, transcriptomic, and protein-protein interaction data along with their merits and demerits (Nesvizhskii, 2014; Wang et al., 2014; Wang and Zhang, 2014; Ruggles et al., 2017). Transcriptomic data has been previously used for functional annotation of identified proteins and determining proteins interactions leading to protein complexes. This is based on a transcriptome-interactome correlation mapping strategy, i.e., the expression profiles of interacting protein coding genes are correlated and this knowledge can be leveraged to predict protein-protein interactions (Figure 4; Ge et al., 2001; Jansen et al., 2002). This methodology has been extended to also predict protein complexes (Zhang et al., 2004; Ruggles et al., 2017; Will and Helms, 2019). Functionally conserved orthologs protein coding genes have similar expression patterns (Chen and Zhang, 2012; Rodriguez-Cruz et al., 2013; Das et al., 2016), and this can be used to predict protein complexes in related non-model species. Furthermore, the evolutionary rates of the interacting protein partners are known to be constrained with respect to each other (Fraser et al., 2003; Qian et al., 2011; Choi and Hannenhalli, 2013; Schoenrock et al., 2017). Together, the transcriptomic data coupled with evolutionary rate analysis derived from genomes can thus help in increasing the confidence in protein-protein interactions (Schoenrock et al., 2017; Hawe et al., 2019), and to a certain extent protein complexes especially when working with new model systems. Protein complexes, which are constituted of multiple protein interactions, can contain fast-evolving proteins and thus have a higher chance of being incompatible in a hybrid cellular environment. Although more work needs to be devoted to harvest the benefits of this integrative approach, molecular evolutionary analysis of transcriptomic and genomic data show promise in studying ecological molecular speciation mechanisms (Stinchcombe and Hoekstra, 2008; Lee et al., 2014; Ravinet et al., 2017). This is specifically useful for systems where molecular and genetic techniques are inadequate to experimentally detect the genetic barriers of gene flow.

## Discussion

In the long term, the goal is to develop experimental and computational tools that can confidently identify the molecular drivers of speciation. These changes can be at the genomic, transcriptomic, or at the proteomic levels. Since Charles Darwin, who illustrated “a tree of life” based on morphology, we have strived to further refine the branches in the “story of life on earth” with the addition of our knowledge about the biochemistries of metabolism and physiology, tissue histology and cellular structures, sub-cellular structures and organization, protein complexes, and with the recent great advances in genomic and transcriptomic sequencing. In addition to genetic incompatibilities and failures in protein interactions leading to a breakdown in protein complexes; protein-DNA and protein-RNA interactions (Lee et al., 2008; Chou et al., 2010; Henault and Landry, 2017; Jhuang et al., 2017) are also known causes of hybrid breakdown, albeit unexplored. These are other directions in which advances in experimental and computational techniques need development.

In addition to identifying the molecular bases of past speciation events, the aim is to see within genomes, proteomes, and protein complexomes the signatures that may create blocks and barriers in future potential evolutionary trajectories. For example, the inner membrane of the mitochondria has one of the highest known protein:lipid ratios, which is proposed to make membrane integrity relatively fragile among biological membranes. It has been observed in yeast that almost all of the known cases of DM incompatibilities are mitochondrial-nuclear incompatibilities, and that there is a highly conserved AAA-ATPase homeostasis machinery essential for maintaining the integrity of the inner membrane (Francis and Thorsness, 2011; Lee et al., 2017). This suggests that in yeast the complexes of the inner mitochondrial membrane, such as the F1-F0 ATPase, are a “molecular branch point” in the natural history of yeast.

In the longer term, our knowledge about how past speciation events may have occurred in the “molecular story of life,” combined with a new potential ability to identify the potential barriers in possible future evolutionary trajectories, may allow us to bioengineer new species beyond these barriers and create organisms with increased fitness that Mother Nature has failed to create by chance over billions of years of evolution. One possible example is to bioengineer into yeast the vertebrate F1-F0 ATPase c-ring subunits, which only contains eight subunits compared to the 10 c-ring subunits of the yeast F1-F0 ATPase (Song et al., 2018), which means the vertebrate form is more efficient (Watt et al., 2010), and then study using phenotypic, molecular, and proteomic assays under diverse conditions to decipher when protein-protein interactions between the ring complex and associated proteins can increase or decrease compatibility and functionality. Laboratory evolution of hybrids between post-zygotically isolated but closely related species or cells carrying replaced orthologs from closely related species, followed by proteome analysis can help us understand how speciation barrier can be overcome and also the role of protein interactions in maintaining species integrity. For example, laboratory evolution of *S. cerevisiae* cells introgressed with CCM1-*Saccharomyces bayanus* helped discovering the general rules underlying PPR domain evolution (Jhuang et al., 2017). Laboratory experimental evolution refinement may also allow us to create novel yeast species that may outcompete the parental strain in a way never achieved in nature during billions of years of natural selection.

## Author Contributions

KS and J-YL conceived the manuscript. KS, SCS, and J-YL wrote the manuscript and obtained funding. All authors contributed to the article and approved the submitted version.

### Conflict of Interest

The authors declare that the research was conducted in the absence of any commercial or financial relationships that could be construed as a potential conflict of interest.

## Supplementary Material

Supplementary Figure 1 Transcriptional regulation diverges quickly between closely related species that often leads to mis-regulated gene expression in hybrids. mRNA abundance is regulated by the binding of *trans*-factors (mainly Transcription Factors) to *cis*-regulatory elements, where mutations in either of them can affect the mRNA abundance. **(A)** Ancient related species well adapted to a common ancient environment can rapidly evolve to adapt to different new environments *via* mutations that alter mRNA transcription levels and/or mRNA stability. **(B)** The ancient parents displayed hybrid compatibility, where the hybrid mRNA regulatory networks achieved the proper amount of protein production well adapted to their common ancient environment. **(C)** The rapidly evolved incipient species display hybrid incompatibility and are not well adapted to either of the current environments. The mixed regulation within the hybrids yields an average level of mRNA leading to the improper amount of protein that is not well suited for either current environment.
