# Supplementary figures and images for: Protein Complexes Form a Basis for Complex Hybrid Incompatibility
Source: Front Genet. 2021 Feb 9;12:609766. doi: 10.3389/fgene.2021.609766 (PMC7900514; doi:10.3389/fgene.2021.609766)

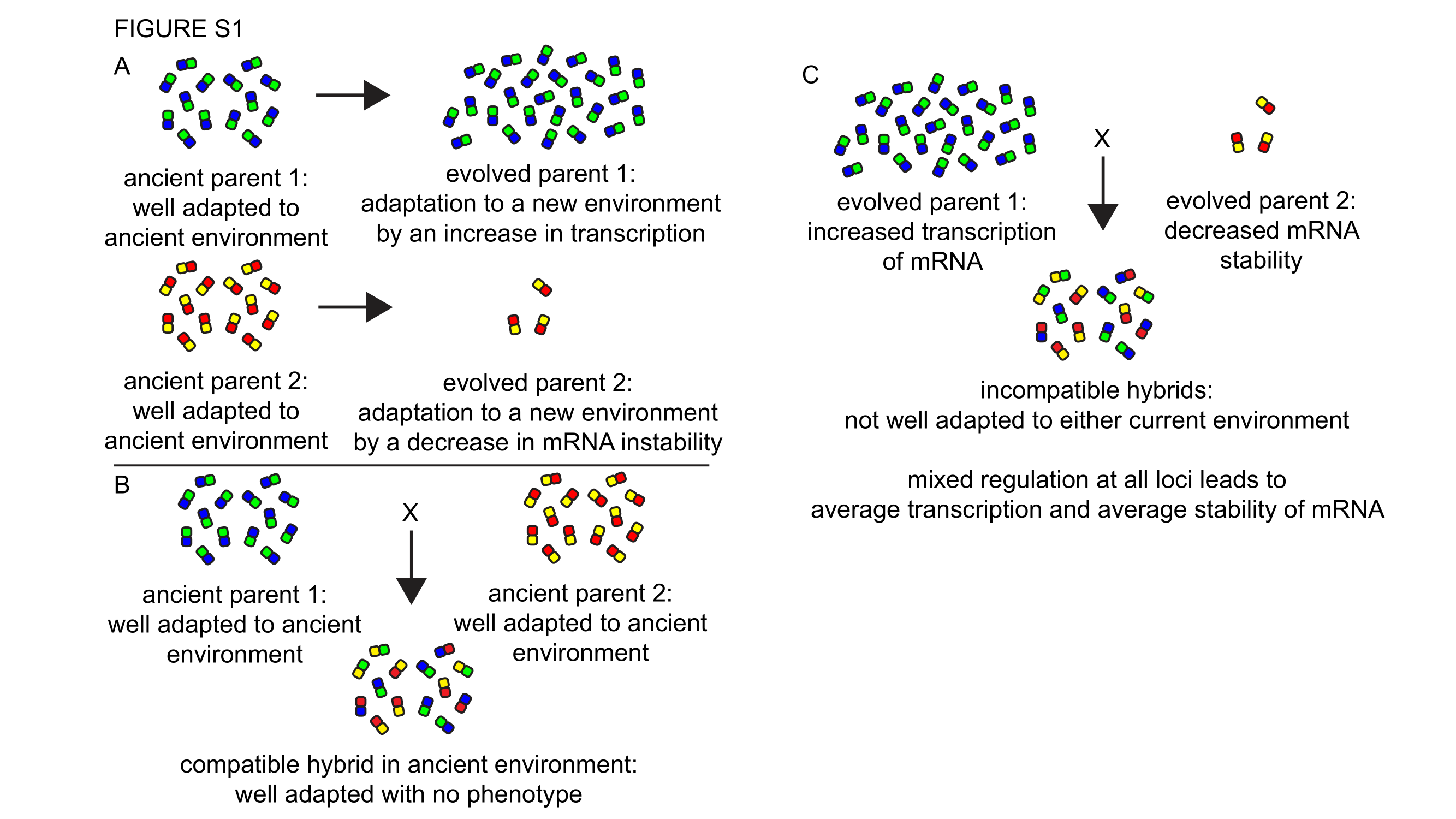

Supplement: Supplementary Figure 1 — Transcriptional regulation diverges quickly between closely related species that often leads to mis-regulated gene expression in hybrids. mRNA abundance is regulated by the binding of trans-factors (mainly Transcription Factors) to cis-regulatory elements, where mutations in either of them can affect the mRNA abundance. (A) Ancient related species well adapted to a common ancient environment can rapidly evolve to adapt to different new environments via mutations that alter mRNA transcription levels and/or mRNA stability. (B) The ancient parents displayed hybrid compatibility, where the hybrid mRNA regulatory networks achieved the proper amount of protein production well adapted to their common ancient environment. (C) The rapidly evolved incipient species display hybrid incompatibility and are not well adapted to either of the current environments. The mixed regulation within the hybrids yields an average level of mRNA leading to the improper amount of protein that is not well suited for either current environment. [file Image_1.tiff]
